# Liraglutide Treatment Ameliorates Neurotoxicity Induced by Stable Silencing of Pin1

**DOI:** 10.3390/ijms20205064

**Published:** 2019-10-12

**Authors:** Marzia Bianchi, Valentina D’Oria, Maria Rita Braghini, Stefania Petrini, Melania Manco

**Affiliations:** 1 Research Area for Multi-factorial Diseases, Obesity and Diabetes, Bambino Gesù Children’s Research Hospital, IRCCS (Istituto di Ricovero e Cura a Carattere Scientifico), viale di San Paolo 15, 00146 Rome, Italy; marzia.bianchi@opbg.net; 2 Confocal Microscopy Core Facility, Research Laboratories, Bambino Gesu’ Children’s Research Hospital, IRCCS (Istituto di Ricovero e Cura a Carattere Scientifico), viale di San Paolo 15, 00146 Rome, Italy; valentina.doria@opbg.net (V.D.); stefania.petrini@opbg.net (S.P.); 3 Molecular Genetics of Complex Phenotypes Research Unit, Bambino Gesù Children’s Research Hospital, IRCCS (Istituto di Ricovero e Cura a Carattere Scientifico), viale di San Paolo 15, 00146 Rome, Italy; mariarita.braghini@opbg.net

**Keywords:** Alzheimer’s disease, brain insulin resistance, brain glucotoxicity, Pin1, type 2 diabetes, type 3 diabetes, 2-deoxy-d-glucose, methylglyoxal, liraglutide

## Abstract

Post-translational modulation of peptidylprolyl isomerase Pin1 might link impaired glucose metabolism and neurodegeneration, being Pin1 effectors target for the glucagon-Like-Peptide1 analog liraglutide. We tested the hypotheses in Pin1 silenced cells (SH-SY5Y) treated with 2-deoxy-d-glucose (2DG) and methylglyoxal (MG), stressors causing altered glucose trafficking, glucotoxicity and protein glycation. Rescue by liraglutide was investigated. Pin1 silencing caused increased levels of reactive oxygen species, upregulated energy metabolism as suggested by raised levels of total ATP content and mRNA of SIRT1, PGC1α, NRF1; enhanced mitochondrial fission events as supported by raised protein expression of FIS1 and DRP1. 2DG and MG reduced significantly cell viability in all the cell lines. In Pin1 KD clones, 2DG exacerbated altered mitochondrial dynamics causing higher rate of fission events. Liraglutide influenced insulin signaling pathway (GSK3b/Akt); improved cell viability also in cells treated with 2DG; but it did not revert mitochondrial dysfunction in Pin1 KD model. In cells treated with MG, liraglutide enhanced cell viability, reduced ROS levels and cell death (AnnexinV/PI); and trended to reduce anti-apoptotic signals (BAX, BCL2, CASP3). Pin1 silencing mimics neuronal metabolic impairment of patients with impaired glucose metabolism and neurodegeneration. Liraglutide rescues to some extent cellular dysfunctions induced by Pin1 silencing.

## 1. Introduction

The Prolyl isomerases (Peptidylprolyl isomerases, PPIases) are a class of enzymes that catalyze the *cis*/*trans* isomerization of the peptide bond between the preceding amino acid and the proline (Pro) residue [1,2,3,4]. The PPIases modulate their stability, enzyme activities and subcellular localization by catalyzing conformational changes of their substrates [3,5,6]. 

The Peptidylprolyl *cis/trans* isomerase NIMA-interacting 1 (Pin1) belongs to one of the three classes of PPIases. Main distinctive feature of Pin1 is that its substrates are phosphorylated by proline-directed kinases. Indeed, Pin1 requires that the Serine (Ser) or the Threonine (Thr) that precede Pro residue are phosphorylated to ensure catalysis [3,7]. Pin1 modulates key proteins involved in cellular processes such as mitosis, neuronal differentiation and metabolism. Dysfunctional expression of Pin1 causes deregulation of Pin1 substrates, a phenomenon that is associated with the onset of neurodegenerative and metabolic disorders including type 2 diabetes (T2D) [1,3,8,9,10,11,12].

In brain, Pin1 modulates neuronal differentiation [13,14] and synaptic plasticity [15,16]. Expression level of Pin1 increases physiologically in neurons during cell differentiation, stays high during the lifespan [13,14], and decreases with aging and in aging-related pathological conditions such as Alzheimer’s disease (AD). Reduced levels of Pin1 cause decreased neuroprotective activity and result in neuronal loss [14]. Pin1 KO (knock out) mice develop neuronal characteristics of premature aging and age-related cognitive decline [14,17,18,19], but also altered insulin signaling in brain, liver and muscle tissues that lead to glucose intolerance and overt T2D [3]. Indeed, Pin1 modulates glycogen synthase kinase-3β (GSK3β) [20] and Akt [21] that are pivotal proteins in insulin signaling. In particular, GSK3β is the crucial enzyme of glycogen synthesis, which plays a key role in regulating blood glucose homeostasis. GSK3β participates to the insulin-signaling cascade through activation of the signal transduction pathway of phosphatidylinositol 3-kinases (PIK3)/Akt [22]. In insulin resistance, GSK3β is increased leading to increased blood glucose. Furthermore it is one of the key factors that mediate islet β cells apoptosis and, therefore, is closely related to insulin deficiency. In the brain, excessive activation of GSK3β promotes abnormal hyper phosphorylation of tau protein, aggravates degeneration of neurons, interferes with normal synaptic plasticity, and accelerates AD pathology process in AD patients [22]. Anomalous regulation of GSK3β activity has been associated to neurodegenerative disorders like AD, and diabetes [1,20,22]. Human Pin1 KD (knock down) cells show reduced level of the GSK3β inactive form (phosphorylated GSK3β pGSK3β) [20].

AD and T2D share molecular, biochemical, pathophysiological and metabolic dysfunctions, such as peripheral and central insulin resistance, chronic low grade inflammation, enhanced oxidative stress, DNA damage, mitochondrial dysfunction and reduced insulin secretion. Owing to brain insulin resistance, reduced glucose metabolism and insulin deficiency, AD is termed as ‘type 3 diabetes’ (T3D) [22,23].

Mitochondria have pivotal role in cellular energy supply. As dynamic organelles, mitochondria undergo continuous fissions and fusion. Fission events are regulated by dynamin-related protein (DRP1), while fusion events by mitofusins, MFN1 and MFN2 and OPA1 (optic atrophy type 1) that are dynamin-related GTPases. Mitochondrial dysfunctions are early events in the pathogenesis of insulin resistance, T2D and AD [24] and trigger the apoptotic cascade induced by oxidative stress and redox imbalance. 

2-deoxy-d-glucose (2DG) and methylglioxal (MG) are metabolites that may compromise energy efficiency of the neuronal cell causing impaired glycolysis and, in that, mimicking metabolic dysfunction of neurons in AD and other conditions of brain insulin resistance. Altered glycolysis is constantly associated with neurodegeneration and memory loss [25]. 2DG, a non-metabolizable glucose, inhibits glycolysis and induces the compensatory utilization of alternative energetic substrates such as ketone bodies that reduce cellular oxidative stress and ensure neuronal survival in rodent model of Parkinson’s disease (PD) and ischemia [26,27,28]. Nevertheless, in different experimental models 2DG has been found to cause cellular stress [29], altered DNA repair activity, but affected also production of reactive oxygen species (ROS) scavengers or antioxidants hence reducing metabolically active cells [30]. In 6-month female mouse model of AD, 7-week dietary exposure to 2DG was associated with oxidative stress but also non-amyloidogenic processing of the amyloid precursor protein (APP), reduced production of amyloidβ (Aβ) oligomers, and increased expression of neurotrophins [27]. 

MG, a dicarbonyl compound, is a side-product of glycolysis that causes cellular damage trough cross-linking and glycation of proteins [31]. MG is a precursor of advanced glycation end-products (AGEs) that increases intracellular oxidative stress by augmenting production of reactive oxygen species (ROS) [32], and AGEs that contribute to cross-linking of proteins in Aβ deposits. In doing so, it acts as mitochondrial toxicant in neurodegenerative disorders that impairs energy balance and causes apoptosis. In neuroblastoma cells [33] and cultured rat hippocampal neurons [34], MG up-regulates expression of apoptotic markers [31]. Elevated levels of MG are found in cerebrospinal fluid of patients with neurodegenerative disorders, e.g., those with AD [31], wherein induces hyper phosphorylation of Tau by enhancing kinase activities (i.e., GSK3β) and/or reducing levels of phosphatase (i.e., those of protein phosphatase 2A) [31]. In vitro and in vivo experimental evidence demonstrates that exogenous administration of MG induces insulin resistance and other metabolic features of patients with T2D, such as hypercholesterolemia and microvascular degeneration [35]. 

The glucagon-like peptide-1 (GLP-1) is a gut incretin that stimulates β-cell insulin secretion and likewise as its analog liraglutide (Lira), crosses the blood–brain barrier where acts as growth factor to enhance neurogenesis and synaptogenesis [36,37]. GLP-1 and GLP-1 analogs are effective in restoring peripheral insulin sensitivity and blood glucose homeostasis by stimulating glucose uptake via the activation of PI3K/Akt signaling cascade [38]. Liraglutide is a long-acting analog of GLP-1 approved for the treatment of T2D that has been found to improve cognitive performance, synaptogenesis and mitochondrial biogenesis in animal models of AD [39,40] and PD [41]. Efficacy of liraglutide to ameliorate cognitive impairment in patients with AD has been recently tested in pilot clinical trial [42]. Twenty-six-week liraglutide treatment was associated with an increase of cerebral glucose consumption, even though not statistically significant, but no change in the cognitive score as compared to the placebo. Liraglutide did not prevent Aβ deposition [42]. 

Aim of the present study was (i) to demonstrate that in vitro silencing of Pin1 results in altered energy metabolism (cellular Adenosine triphosphate content, ATP; *NAD-dependent deacetylase* 1 *Sirtuin* 1, *SIRT*1; *Peroxisome proliferator-activated receptor gamma coactivator* 1-alpha, *PGC*1α; *nuclear respiratory factor* 1, *NRF*1) and dysfunctional mitochondrial dynamics (Fission: DRP1, FIS1; fusion: MFN1 and OPA1); (ii) to test the efficacy of liraglutide to counteract Pin1-silencing-induced-neurotoxicity. To accomplish the aims of the study, we investigated mitochondrial proteins involved in fission and fusion; markers of insulin signaling pathway (GSK3β and Akt), cell viability, and response to oxidative stress (ROS production) and markers of apoptosis (BCL-2 associated X protein, *BAX*; B-cell lymphoma 2, *BCL*2; *Caspase 3, CASP*3*; Tumor Protein 53, TP*53). Altered energy metabolism and cellular stress were induced by treatment with 2DG and MG.

## 2. Results

### 2.1. In Vitro Modeling of Human Neurodegeneration 

**Figure 1:**
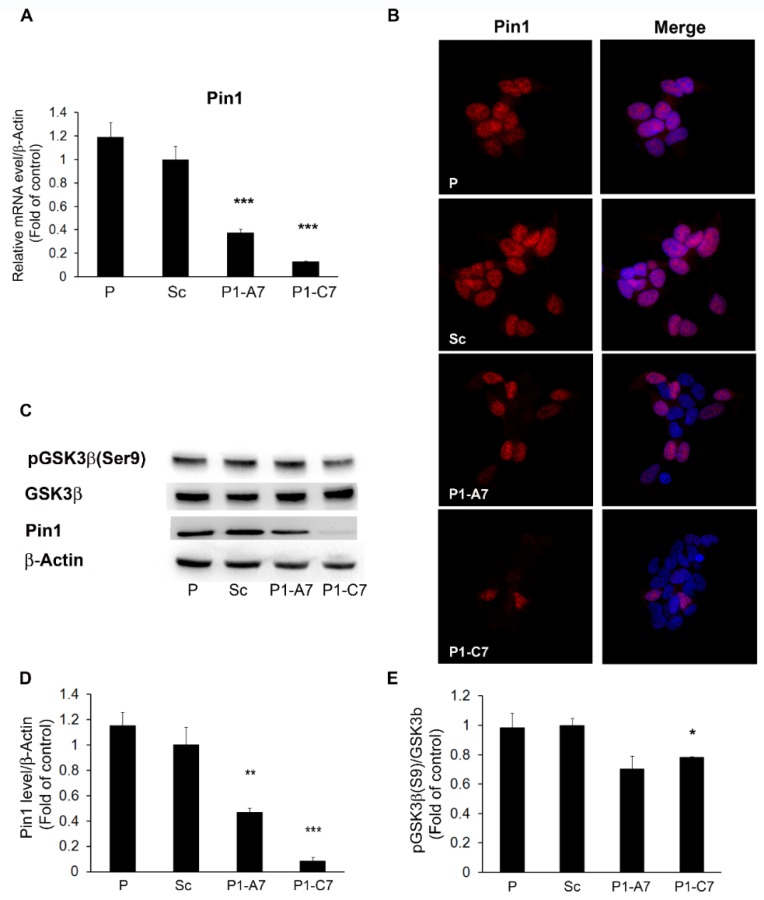
In vitro model of engineered neuroblastoma cells: SH-SY5Y as parental cells (P), Scramble (Sc) and silenced Pin1 clones (P1-A7 and P1-C7). (**A**) Quantitative evaluation of Pin1 mRNA expression level by qPCR. (**B**) Representative fluorescence images of Pin1 in P, Sc and Pin1 KD clones. Red: Anti-Pin1; nuclei are counterstained with Hoechst 33342 (blue color) and combined images are shown. Magnification Images 60×. (**C**) Representative western blots of Pin1, pGSK3β(S9) and GSK3β expression. Lane 1: Parental cells SH-SY5Y, not transfected; lane 2: SH/Scramble cells as negative control for silencing; lane 3: Stable P1-A7 silenced clone; lane 4: P1-C7 silenced clone. (**D**) Quantitative evaluation of Pin1 protein expression level. (**E**) Expression of pGSK3β(S9) respect to GSK3β. β-Actin expression levels were used as loading reference. The data are representative of three independent experiments and are shown as mean values ± SD. * *p* < 0.05; ** *p* < 0.01, *** *p* < 0.001 vs Scramble clone.

In an in vitro system of human neuroblastoma cell line, SH-SY5Y, we silenced the expression of *Pin*1 gene by RNA interference technique. Real-time quantitative PCR (qPCR) and protein expression level revealed two Pin1 knock down (KD) stable clones with about 60% (P1-A7) and 90% (P1-C7) efficacy of silencing, respectively (Figure 1A–D). The stable transfection of the scramble (Sc) clone was used as negative control for silencing expression and SH-SY5Y cells were the parental cells (P). Figure 1B showed silenced Pin1 lines at confocal microscopy. Figure 1C,D reported western blots and relative quantification confirming Pin1 silencing of about 60% and 90%, respectively.

Protein levels of GSK3β and its inactive form pGSK3β(Ser9) confirmed goodness of the model. In Pin1KD clones there was a significant reduction of pGSK3β(Ser9) expression, while levels of total GSK3β were unchanged. The pGSK3β to GSK3β ratio was reduced by 28% and 22% in the two Pin1 KD clones, respectively (Figure 1C,E). 

#### 2.1.1. Pin1 Silencing Influences Cell Energy Metabolism

**Figure 2:**
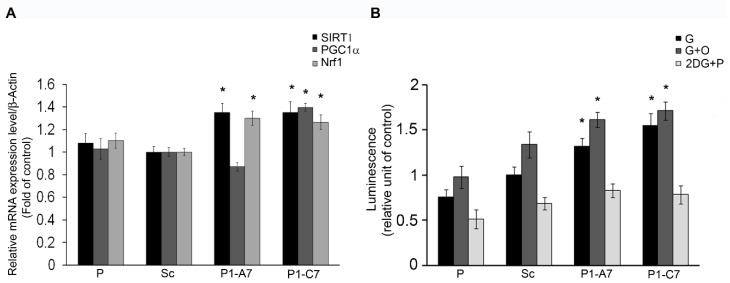
Effects of Pin1 silencing on cell energy metabolism. (**A**) mRNA expression levels of *SIRT*1, *PGC*1a and *NRF*1. (**B**) To measure the total ATP content, cells were incubated for 2 h with either 10 mM d-glucose (G), 10 mM d-glucose plus 2.5µg/mL oligomycin (G + O), or 50 mM 2-deoxy-D-glucose plus 5 mM sodium pyruvate (D + P). Results are expressed as mean values ± SD. * *p* < 0.05 vs. Scramble clone.

Cell energy metabolism was up-regulated as demonstrated by increased levels of the tested markers in growing condition (Figure 2A). Pin1 KD clones showed up-regulated *SIRT*1 (~35% for both) and *NRF*1 mRNA levels (29% and 27%, respectively) (Figure 2A). The P1-C7 clone had significantly higher *PGC*1α mRNA level (~40%) as compared with P, Sc and P1-A7 clones. 

ATP production was measured in cells cultured with glucose (G); glucose with oligomycin (G + O) to measure ATP production exclusively from the glycolytic pathwhay since olygomicin A inhibits ATP synthase; 2DG with pyruvate (2DG + P) to measure exclusive mitochondrial ATP synthesis since pyruvate is an important energy supply. By doing so we were able to discriminate ATP production from different pathways [43]. 2DG caused cell deprivation of ATP. Both Pin1 KD clones showed increased ATP content in presence of glucose (G) and glucose and oligomycin (G + O), while no difference in mitochondrial ATP was observed in controls (Figure 2B).

#### 2.1.2. Pin1 in Insulin Signaling Cascade. Liraglutide Restored Impaired GSK3β and Akt Activities 

To evaluate the capability of Lira treatment to rescue insulin signaling in the engineered model, we assessed protein expression level of GSK3β and Akt following treatment with the GLP-1 analog. The best working concentration of Lira (100 nM Lira for 5 h in serum free medium) was established by estimating cell viability (MTT assay) at different doses (Figure S1A). In this experimental condition, the P cells showed the significant increase of cell viability by ~20% (Figure S1A).

**Figure 3:**
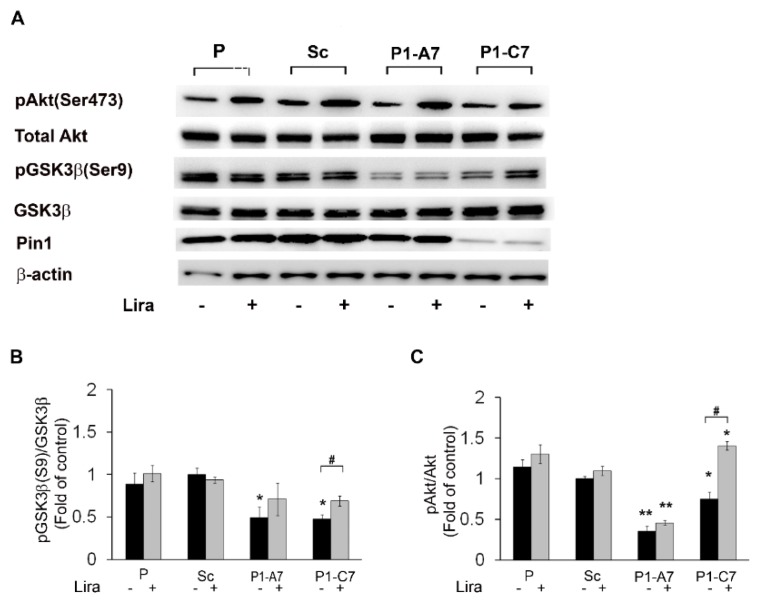
Lira restored protein expression level of GSK3β and Akt phosphorylated forms in Pin1 KD clones. (**A**) Representative western blots experiments of Pin1, pGSK3β(Ser9), GSK3β, pAkt(Ser473), and Akt expressions. (**B**) Quantitative evaluation of pGSK3β(Ser9) versus GSK3β expression. (**C**) Quantitative evaluation of pAkt(Ser473) versus total Akt expression. β-Actin expression levels were used as loading reference. Data are representative of three independent experiments and are shown as mean values ± SD. * *p* < 0.05; ** *p* < 0.01 vs Scramble clone. # *p* < 0.05 baseline versus treatment.

Treatment with 100 nM Lira restored level of pGSK3β(Ser9) in the most silenced P1-C7 KD clone respect to controls (Figure 3A,B) and enhanced phosphorylation of Akt(Ser473) in the all samples and more significantly in the P1-C7 KD clone (Figure 3A,C).

#### 2.1.3. Liraglutide Enhanced Cell Viability in Presence of Impaired Glycolysis 

First we evaluated the response of KD clones to defective/impaired glycolysis. Cells were treated with 2DG and cell viability (MTT assay) and ROS production were assessed. The best working concentration of 2DG (50 mM) was established in P cells starved for 5 h in serum free medium and then incubated with different concentration of 2DG for 1 h (Figure S1B). At dose of 50 mM, P cells showed a statistical significant reduction in cell viability of about 30%. 

**Figure 4:**
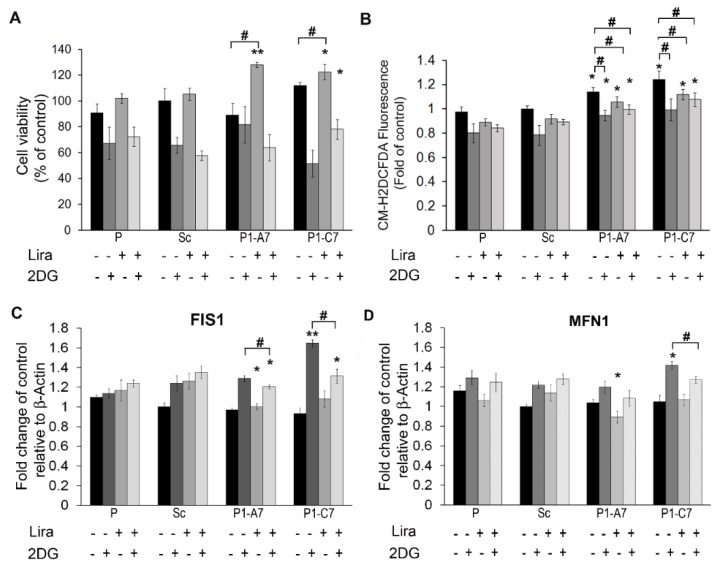
Lira treatment alleviated toxic effect of impaired glycolysis by 2DG. (**A**) Cell viability was evaluated by MTT assay in cells treated with or without 100 nM Lira for 5 h and then treated with/without 50 mM 2DG for 1 h. Cell viability is expressed as percentage respect to that of Sc. (**B**) ROS levels were evaluated in cells treated with or without 100 nM Lira for 5 h and then treated with/without 50 mM 2DG for 1 h. ROS production was measured by CM-H2DCFDA fluorescence intensity and expressed as fold change respect to Sc. (**C**,**D**) Quantitative evaluation of mRNA expression level of *FIS*1 and *MFN*1 genes, respectively, in cells treated with or without 100 nM Lira for 5 h and then treated with/without 50 mM 2DG for 1 h. Results are expressed as mean values ± SD. * *p* < 0.05; ** *p* < 0.01 vs. Scramble. # *p* < 0.05 within the clone.

Figure 4A,B show cell viability and ROS productionof P, Sc and Pin1 KD lines in the different experimental conditions. 

Cells were treated with or without 100 nM Lira for 5 h in serum free medium and then cells were treated with/without 50 mM 2DG for 1 h. Lira treatment improved cell viability in all the cells and increase was statistically significant in Pin 1 KD clones (Figure 4A). After short starvation (5 h), ROS production increased significantly in Pin1 KD clones (Figure 4B) and 2DG, Lira and their combination reduced them in Pin1 KD clones respect to Sc. 

As to dysfunctional mitochondrial network, we found significantly higher mRNA expression levels of FIS1 in the P1-C7 KD clone treated by 2DG respect starvation, while Lira treatment alone and combined treatment with 2DG decreased FIS1 expression levels significantly in both KD clones (Figure 4C). 2DG treatment up-regulated significantly MFN1 expression level in P1-C7 KD clone respect to controls, while Lira treatment in presence of 2DG was effective to lower it (Figure 4D).

**Figure 5:**
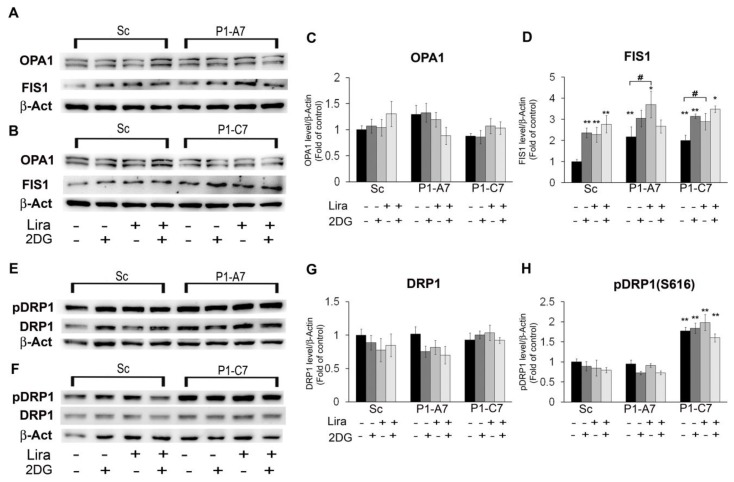
Lira treatment did not rescue enhanced mitochondrial fission in P1-C7 KD clone. Mitochondrial markers of fission and fusion events in cells treated with or without 100 nM Lira for 5 h and then treated with/without 50 mM 2DG for 1 h**.** (**A**,**B**) Representative western blots of OPA1 and FIS1. (**C**,**D**) Quantitative determination of OPA1 and FIS1 protein expressions. (**E**,**F**) Representative western blots of DRP1 and pDRP1(S616) protein expressions. (**G**,**H**) Quantitative determination of protein expression of DRP1 and pDRP1(S616), respectively. *β-*Actin expression levels were used as loading reference. Data are representative of three independent experiments and are shown as mean values ± SD. * *p* < 0.05; ** *p* < 0.01 vs Scramble. # *p* < 0.05 within the clone respect to starvation.

In Figure 5A–H, we reported protein levels of mitochondrial fission and fusion markers. 

Pin1 silencing did not affect mitochondrial fusion events (Figure 5A–C). Conversely, it lead to increased fission events. Both starved Pin1 KD clones showed a statistical increase in FIS1 protein levels respect to Sc. Treatment with Lira, 2DG, and their combination increased further FIS1 protein levels in Sc and Pin1 KD clones respect to the starved condition (Figure 5A,B,D). Neverthless, we found that the combined treatment with Lira and 2DG did not reduce fission events (Figure 4C). Figure 5E–H shows levels of DRP1 protein and its phosphorylated form pDRP1(S616). The GTPase DRP1 mediates mitochondrial fission after its phosphorylation in correspondence of Ser616 residue. This post translation modification allows DRP1 to translocate from cytosol to mitochondrion. We found no difference in DRP1 protein levels regardless of cell lines and treatment. The pDRP1(S616) levels increased significantly in P1-C7 KD clone respect to Sc in all the experimental conditions. In this case too, Lira did not reduce fission events but conversely it increased significantly them alone and in combination with 2DG. 

**Figure 6:**
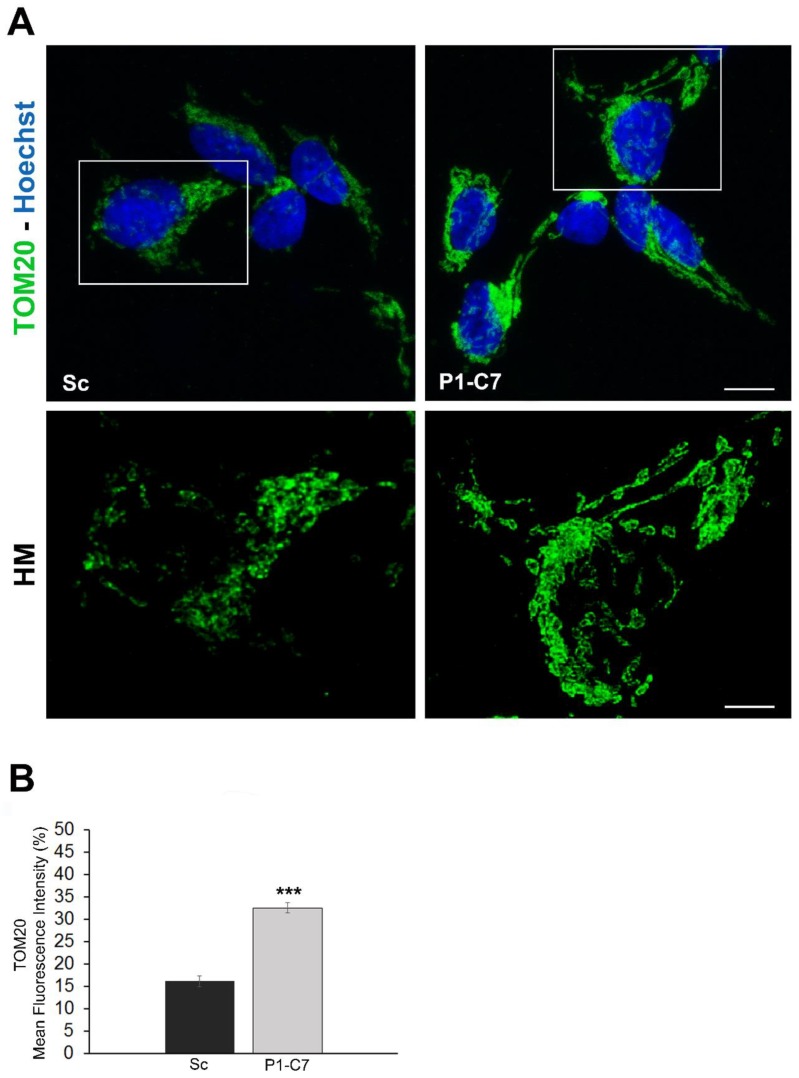
(**A**) Upper panel: Confocal microscopy images (Z-reconstruction) of TOM20 (green) immunofluorescence in Scramble (Sc) and silenced Pin1 (P1-C7) cells showing changes in mitochondrial amount and distribution. Nuclei are counterstained with Hoechst 33342 (blue). Bar: 10 μm. Lower panel: High magnification (HM) of deconvolved z-stack images highlighted in *insets*, in which a higher mitochondria amount, particularly in perinuclear area and in cellular extensions, was observed in silenced Pin1 cells. Bar: 5 μm. (**B**) Mean fluorescence intensity of TOM20 in Scramble (Sc) and silenced Pin1 (P1-C7) cells. Data are presented as the mean ± SEM (*** *p* > 0.001).

Confocal imaging of cells immunolabelled with a TOM20 antibody, a marker of the outer mitochondrial membrane, revealed a significant higher amount and different distribution around nuclei and in cellular extensions of the mitochondrial network in the cytoplasm of Pin1 KD cells respect to Sc in condition of starvation (Figure 6).

#### 2.1.4. Liraglutide Enhances Cell Viability, Anti-Apoptotic Signals and Reduced ROS in Cells Treated with MG 

**Figure 7:**
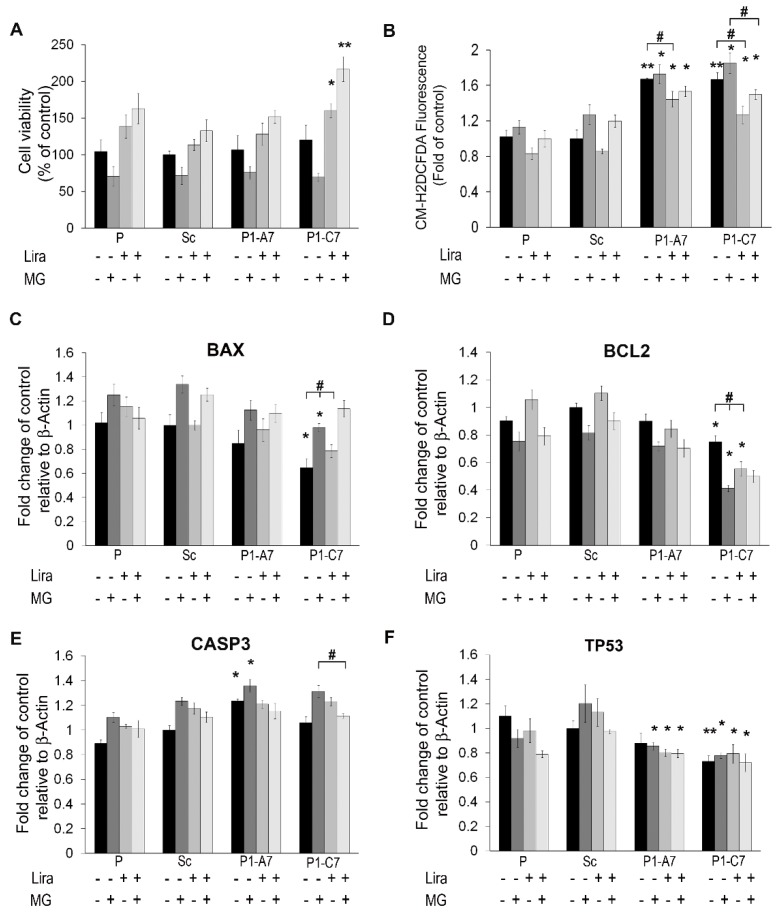
Lira treatment alleviated MG toxic effect. (**A**) Cell viability was evaluated in cells treated with/without 100 nM Lira for 5 h and then treated with/without 600 μM MG for 24 h. Cell viability was measured as percentage of viability in the scramble clone (Sc) by MTT assay. (**B**) ROS production was evaluated in cells treated with/without 100 nM Lira for 5 h and then treated with/without 600 μM MG for 24 h. ROS production was measured by CM-H2DCFDA fluorescence intensity indicated as fold of that in the Sc clone. (**C**–**F**) Quantitative evaluation of mRNA expression level of apoptotic genes: *BAX*, *BCL*2, *CASP*3, *TP*53 in cells treated with/without 100 nM Lira for 5 h and then treated with/without 600 μM MG for 24 h. Results are expressed as mean values ± SD. * *p* < 0.05; ** *p* < 0.01 vs. Scramble. # *p* < 0.05 within the KD clone.

Cell viability was tested in presence of different concentrations of MG for 24 h (Figure S1C). Viability of parental cells was determined by the MTT assay following 5 h starvation and 600 uM MG for 24 h in serum free medium. In this condition, the percentage of metabolically active cells decreased about 40%. Cell viability following starvation and treatment with MG was not different among parental cells and Pin1 KD clones. Treatment with Lira reduced toxicity of MG in all the samples ameliorating cell viability. The best effect was seen in the P1-C7 clone (Figure 7A). Figure 7B shows that starved KD clones had higher ROS production than P and Sc lines. Treatment with MG caused a further significant increase of ROS production in Pin1 KD clones and a trend in P and Sc lines. Lira treatment reduced ROS production in starved cell lines and in those treated with combined Lira and MG.

Hence, we investigated mRNA expression level of apoptotic markers (i.e., *BAX, BCL*2*, CASP*3*, TP*53) following treatment with MG and rescue by Lira (Figure 7C–F). MG up-regulated significantly expression of pro-apoptotic *BAX* mRNA in P lines and all the clones. Lira treatment alone and in combination with MG did not affect *BAX* level. In the P1-C7 KD clone *BAX* mRNA level was significantly lower than in P and Sc cell lines (Figure 7C).

Conversely, MG treatment reduced significantly the anti-apoptotic mRNA levels of *BCL*2 making cells prone to apoptosis. Lira alone and in combination with MG increased levels of *BCL*2 in P and Sc cell lines. In both KD clones Lira did not increase mRNA levels of *BCL*2, while treatment with Lira in presence of MG did it in P1-C7 clone (Figure 7D). MG induced significant up-regulation of mRNA levels of *CASP*3 in all the cell lines. Only in the P1-C7 clone the Lira in combination with MG treatment reduced the mRNA level of *CASP*3 (Figure 7E).

Since MG up-regulates p53 [44], pivotal in glucose metabolism and ROS production [45], we evaluated mRNA expression level of *TP*53 gene, encoding for the tumor suppressor p53 protein, and we found that MG treatment up-regulated levels of *TP*53 mRNA solo in the Sc cell line. Lira treatment alone and in combination with MG decreased levels of *TP*53 mRNA and counteracted MG induced apoptosis in control cell lines. Both Pin1 KD clones showed a lower levels of *TP*53 mRNA under starvation than controls. Combination of MG and Lira did not modulate mRNA expression level of *TP*53.

**Figure 8:**
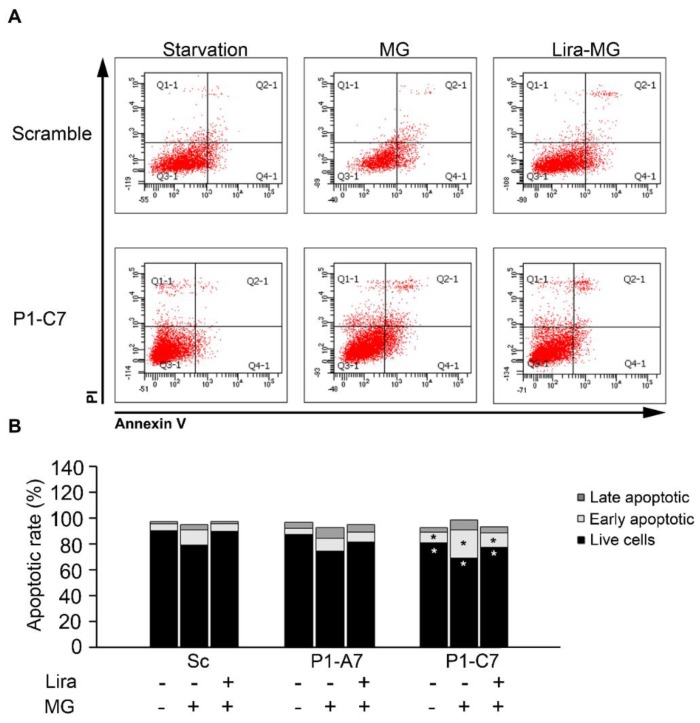
Lira treatment alleviated toxic effect of MG. Apoptotic rate was measured in cells treated with or without 100 nM Lira for 5 h and then treated with/without 600 μM MG for 24 h and cytometry. (**A**) Representative dot plots of annexin V-FITC binding and PI uptake of scramble and P1-C7 KD clone in the three experimental conditions. (**B**) The bar chart depicts the percent distribution of alive (intact), early- and late-apoptotic cells. The percentage of annexin V-positive cells obtained from three separate experiments. Results are expressed as mean values. * *p* < 0.05 vs. Scramble.

In Figure 8A,B cell apoptosis rate measured by flow cytometry using annexin V-FITC/PI double labelling assay was reported in cells treated with or without 100 nM Lira for 5 h followed by exposure with/without 600 μM MG for 24 h. 

Percentages of viable (annexin V-negative/PI-negative, Q3-1 quadrant), early apoptotic (annexin V-positive/PI-negative, Q4-1 quadrant), late apoptotic (annexin V-positive/PI-positive, Q2-1 quadrant) and necrotic cells (annexin V-negative/PI-positive, Q1-1) were evaluated (Figure 8A). Starved P1-C7 KD clone showed lower percentage of alive cells (80.8%) and higher of early apoptotic ones (8.1%) than Sc cells (Sc, 4.9%). MG treatment reduced significantly the percentage of alive cells in the P1-C7 clone (69.2% versus 78.95% in Sc cells), while it increased the percentage of apoptotic ones in all the cell lines as compared to the starvation status. The effect was more evident in P1-C7 clone (21.8% versus 11.9% in Sc cells). Lira in combination with MG increased the percentage of alive cells and reduced the percentage of the apoptotic ones in all the samples respect to the MG treatment. 

## 3. Discussion

Pin1 gene silencing was associated with up-regulated energy metabolism in neuronal cells, with an increased mitochondrial fission and impaired insulin signaling owing to reduced levels of pGSK3β(S9) that was rescued by liraglutide treatment. The drug improved cell viability in the engineered model in presence of 2DG, a compound that impairs glycolysis and, doing so, mimics altered glucose metabolism of neurons in neurodegenerative diseases. In Pin1 KD clones, 2DG caused an exacerbated mitochondrial dynamics characterized by higher rate of fission events. Liraglutide did not revert 2DG-induced mitochondrial dysfunction. Conversely, it enhanced cell viability and anti-apoptotic signals and reduced ROS levels in Pin1 KD clones when exposed to MG, a compound that induces insulin resistance.

In vitro and in vivo evidence supports the capability of liraglutide and other GLP-1 analogs to improve systemic and brain insulin signaling and glucose metabolism, acting as growth factors that improve synaptic plasticity and reduce neurotoxicity. Treatment with GLP-1 analogs is promising for altered glucose homeostasis and cognitive decline of patients with T2D-associated dementia or AD [36,37,39,40,41,42]. 

### 3.1. Silencing of Pin1 and Cell Energy Metabolism

Silencing of Pin1 determines reduced protein level of pGSK3β(Ser9), the inactive form of GSK3β [20]. Inappropriate activation of GSK3β worsens neurodegeneration in patients with AD, brain insulin resistance or T2D [22].

Pin1 acts as energy switch in cancer cells determining the so called “Warburg effect” or “aerobic glycolysis”, i.e., glycolysis becomes the main source of ATP also in aerobic condition [46]. In human glioblastoma cells, the activity of the Pin1 isomerase drives the correct binding of Phosphoglycerate kinase 1 (PGK1) to the outer membrane translocase (TOM) complex allowing its mitochondrial translocation. PGK1 is the first enzyme in the glycolytic pathway that generates ATP [47], while in mitochondria PGK1 acts as a protein kinase regulating the activity of the pyruvate dehydrogenase complex. Pin1 silencing blocks PGK1 that, in turn, inhibits the pyruvate dehydrogenase complex within the Krebs cycle while favors the glycolysis [47,48].

In keeping with the effect of Pin1 silencing on energy metabolism, ATP content was extremely high in both Pin1 KD clones, owing to the enhanced glycolysis. Dealing with neuroblastoma cells, in our study, we used a concentration of 2DG (50 mM) higher than the dose used by McKenzie and co-workers in fibroblasts [43]. An equal dose was used in retinoblastoma cells to distress the glycolytic pathway [49]. 2DG is a competitive inhibitor of glucose not metabolized by hexokinase and, whenever the cause is, any impairment of the glycolysis results in neurodegeneration, brain insulin resistance, memory loss, and dementia [25].

To investigate the effects of Pin1 silencing in energy metabolism we evaluated the mRNA expression level of three genes *SIRT*1, *PGC*1α, and *NRF*1 as markers of cell metabolism and redox signaling pathway, respectively. In both Pin1 KD clones, the reduction of mRNA *Pin*1 levels was counteracted by the up-regulated expression of *SIRT*1 and *NRF*1. The P1-C7 clone showed also the up-regulation of *PGC*1α, master regulator gene of mitochondria biogenesis and antioxidant gene. In that, it seemed that silencing of Pin1 mimics the effects of starvation in stimulating cell metabolism that, we speculate, could be an attempt of the cell at counteracting neurodegeneration. Indeed, SIRT1 is differently regulated in many oxidative-stress-associated diseases i.e., neurodegenerative and cardiovascular disease and increases insulin sensitivity in T2D and aging [50]. SIRT1 triggers cell metabolism by recruiting Akt that phosphorylates and regulates the subcellular cytoplasm/nucleus trafficking of FOXOs transcription factors and by modulating AMP-activated protein kinase (AMPK) phosphorylation [50]. Activation of AMPK promotes insulin sensitivity and mitochondrial biogenesis by increasing PGC1α and NRF1 levels. SIRT1 and AMPK are interdependent and regulate each other’s reciprocally [51]. Pin1 binds the γ subunit of AMPK acting as a negative regulator of AMPK in muscle [52]. Indeed, Pin1 KO mice show higher levels of AMPK phosphorylation than wild-type controls causing up-regulation of PGC1α and NRF1 [52]. Pin1 modulates insulin signaling by modulating Akt, stabilizing the balance between active/stable versus inactive/unstable Akt [21] and GSK3β activities [20]. Moreover, in a mouse model of AD SIRT1 deacetilase activity/protein was increased in brain activating the pathway through which the caloric restriction allows to reduce Aβ deposition [53]. 

### 3.2. Liraglutide Relieves 2DG Induced Neurotoxicity and Enhances Cell Viability in Pin1 KD Clones

Liraglutide was effective to rescue neurotoxicity induced by the silencing of Pin1, raising protein expression of inactive GSK3β (pGSK3β(Ser9)) and phosphorylated Akt [37]. Both kinases are important effectors in the insulin-signaling cascade and the deregulation of the insulin/PI3K/Akt pathway is associated to brain impaired glucose homeostasis and causes tau hyper phosphorylation [22], raised apoptotic stimulus and mitochondrial dysfunction, increased mitochondrial-mediated apoptosis and oxidative stress [54].

Our results confirm previous evidence [32] that liraglutide augments cell viability and this effect was evident especially in Pin1 KD clones. In parental and scramble cell lines, cytotoxicity of 2DG treatment reduced cell viability by about 40%, while reduction of cell viability was even more evident in the most silenced P1-C7 clone. Liraglutide was cytoprotective against 2DG treatment ameliorating cell viability in the silenced clones.

Then, we evaluated the effect of Pin1 silencing on oxidative stress and the protective role of liraglutide treatment over 2DG treatment. The net balance between the antioxidant systems and the redox signaling ensures ROS homeostasis and, in turn, cell viability under stress stimuli [55]. Both high concentration of 2DG (50 mM) and starvation [49] induce ROS production causing cytotoxicity. We observed that both Pin1 KD clones had higher ROS production under starvation respect to controls. 2DG treatment reduced ROS production in all cell lines [56] by blocking glycolysis and favoring metabolism through the pentose phosphate pathway [49], confirming a neuroprotective role for the 2DG [26,27,28]. Liraglutide reduced ROS production as previously demonstrated [57]. Liraglutide treatment followed by 2DG treatment was effective to reduce ROS production in all the samples respect to the starved cells. 

Balancing fission and fusion events is essential for mitochondrial network integrity. Fusion events are regulated by the co-operative action of the large dynamin-related GTPases known as mitofusins, MFN1 and MFN2 at the outer mitochondrial membrane and OPA1 at the inner mitochondrial membrane. Fission events of the outer mitochondrial membrane are regulated by the enzyme DRP1. These proteins responsible for the fission and fusion events are regulated by proteolysis and post-translational modifications [58]. The DRP1 localization at mitochondria is transient. Indeed, the DRP1 translocation from cytosol to mitochondrion is dependent from the phosphorylation of Ser616 residue and its anchoring to the membrane is tightly regulated by the recruitment of outer membrane protein adaptors, such as FIS1 [59].

Loss of integrity in the mitochondrial network is common feature of neurons in T2D and neurodegenerative disorders [24]. Fusion events are often associated with an attempt at protecting mitochondrial functions [60]. Fission events not only ensure a proper mitochondrial number to daughter cells during cellular division, but also may help to isolate damaged mitochondria promoting removal by mitophagy, and to facilitate apoptosis in condition of high cellular stress [60]. A direct AMPK activation through a family of small molecules, although in the absence of mitochondrial damage, is sufficient to induce mitochondrial fission. Thus, AMPK becomes a fundamental and direct regulator of mitochondrial dynamics and homeostasis [61]. Pin1 has a negative role in regulating AMPK activation [52].

In our in vitro model, Pin1 silencing affected mitochondrial dynamics unbalancing toward fission events as shown by qPCR and protein levels. In the starved P1-C7 clone, the mitochondrial network was altered due to higher number of mitochondria and different distribution respect to scramble clone. The inhibition of mitochondrial ATP synthesis (i.e., 2DG treatment), triggers mitochondrial fragmentation by increasing mitochondrial fission rates and/or decreasing mitochondrial fusion rates [61]. Indeed, 2DG treatment further unbalanced the mitochondrial dynamics toward fission events in both KD clones especially in P1-C7 cells as evident by qPCR and protein level of *FIS*1 gene, respectively. Also the protein level of pDRP1(S616) was in keeping with enhanced fission events in condition of starvation and in the different experimental conditions. While liraglutide rescued 2DG toxic effect, downregulating *FIS*1 and *MFN*1 mRNA level in both KD clones, no decrease was observed in the protein levels. Increased fission rate induced by liraglutide might represent a mechanism to trigger mitophagy and remove damaged mitochondria.

### 3.3. Liraglutide Reduces MG Neurotoxicity: Impaired Cell Viability, ROS Production and Cell Apoptosis

MG is a highly reactive metabolic intermediate of glycolysis that mimics in vitro chronic glucotoxicity induced by protein glycation and formation of AGEs as it occurs in neurodegenerative diseases and T2D. In our investigation, MG reduced cell viability with no difference among controls and Pin1 KD clones. Liraglutide treatment alone and in combination with MG was cytoprotective enhancing cell viability as previously demonstrated [32,37]. Of note, this effect was preeminent in the P1-C7 KD clone. Starved Pin1 KD clones showed ROS production higher than controls and MG induced significantly higher ROS levels in the former clones as expected [62]. Liraglutide treatment decreased ROS levels also in MG treated Pin1 KD clones.

With regard to apoptosis, Pin1 plays a role at mitochondrial level modulating transcription and stability of p53 among other molecules [63]. p53 protein regulates many cellular pathways, such as energy metabolism, genomic stability, and damaging and antioxidant functions by controlling either cytostatic or cytotoxic responses to various cellular stressors. p53 exerts a transcriptional role in the nucleus where it induces pro-oxidant genes or controls expression of antioxidant genes, hence promoting apoptosis, while it acts in the cytosol as tumor suppressor that lead to cell death [55]. Depending on the localization of Pin1 within the cell, the isomerase can modulate p53 responses either triggering mitochondrial translocation of p53 in the cytoplasm or increasing the p53 retention in the mitochondrion [64]. During stress-response signaling, a key transcription factor signal transducer and activator of transcription 1 (STAT1) induces the apoptosis by up-regulating p53 and its transcriptional activity on pro-apoptotic genes such as BAX [44]. After cis/trans isomerization of p53 by Pin1, upon stress induction, Pin1 and p53 act in concert to promote apoptosis by activating the effector BAX protein [65]. This coordinated interaction compromises the integrity of the outer mitochondrial membrane leading to the cascade reactions of mitochondrial-mediated apoptosis [65]. Accordingly, mRNA expression of BAX is downregulated in Pin1 silenced cells after stress stimulus [66]. In cancer cells, it has been observed that Pin1 enhances conformational stabilization and cell death resistance capability of BCL2, an anti-apoptotic effector that in turn inhibits apoptosis via inactivation of BAX [48]. 

In starved Pin1 KD clones, *BAX* and *BCL2* mRNAs were down-regulated, while treatment with MG induced the up-regulation of *BAX* and the down-regulation of *BCL*2 mRNA level, respectively. Liraglutide up-regulated *BCL*2 mRNA expression level in controls but not in Pin1 KD clones. Nevertheless, in the P1-C7 clone it was protective against MG-induced apoptosis, up-regulating mRNA expression level of *BCL*2. Both Pin1 KD clones showed lower *TP*53 mRNA (encoding for p53 protein) than controls in all the experimental conditions and no treatment was effective to modulate *TP*53 mRNA level. 

MG treatment was associated with higher levels of *CASP*3 mRNA level in silenced clones and liraglutide was effective to rescue it in the P1-C7 clone. 

Silencing of Pin1 up-regulated in general cell energy metabolism and enhanced susceptibility to oxidative stress causing redox imbalance by virtue of Pin1 modulation of AMPK. Pin1 silencing influenced also p53 signal transducer and expression level of apoptotic effectors such as *BAX* and *BCL*2, while the expression level of *CASP*3, downstream in the apoptotic signaling cascade, was less affected. Flow cytometry experiments confirmed the susceptibility of Pin1 KD clones to apoptosis induced by stressor such as MG that was particularly toxic for the most silenced clone and the rescue capability of liraglutide. Our findings confirm that liraglutide exerts some neuroprotective effects.

Liraglutide binds GLP-1 receptors, widely expressed in neuronal cells, a coupled G-protein-receptor, from which G-protein beta-gamma subunits dissociates intracellularly to activate PI3K, resulting in activation of Akt, that in turn phosphorylates and suppress GSK3β activity [67]. In our study, the GLP-1 analog at a dose of 100 nM compensated neurotoxicity induced by stable silencing of Pin1 on the PI3K/Akt transduction pathway. Along this pathway, Pin1 binds and/or modulates important kinases [1,21]. Liraglutide promoted cytoprotection against 2DG and MG by improving cell viability, reduced redox imbalance and MG induced apoptosis in cells affected by persistent mitochondrial dysfunction [32,37]. 

## 4. Materials and Methods 

### 4.1. Cell Lines and Chemicals

SH-SY5Y human neuroblastoma cell line was kindly provided by Dr. Rosalba Carrozzo at the Bambino Gesù Children’s Hospital (Rome, Italy). SH-SY5Y cells were grown in DMEM-F12 medium (Euroclone, Pero (Mi), Italy, ECM0095L) supplemented with 10% FBS (Euroclone, ECS0180L), 1% penicillin/streptomycin (Euroclone, ECB3001D) at 37 °C in a humidified 95% air and 5% CO2 atmosphere. The cells were sub-cultured when 80%–90% confluent and seeded at 1:5 ratio. Media was changed every 4–5 days.

SH-SY5Y cells and stable clones were seeded on 96-well plates coated with Matrigel Basement Membrane Matrix (Corning, New York, NY, USA, 354234) at density of 4 × 10^4^ cells per well for 24 h.

### 4.2. In Vitro Treatment 

Liraglutide (Selleckchem, Houston, TX, USA, S8256). The peptide was reconstituted in Gibco Water for Injection for Cell Culture (Thermo Fischer Scientific, Waltham, MA, USA, A1287301) to a concentration of 1 mM, aliquoted and stored at −20 °C until used. Liraglutide working stock preparations were diluted in serum-free culture medium to different nM concentrations. In experiments, liraglutide was used at 100 nM concentration. 

2-deoxy-D-glucose (Sigma Aldrich, St. Louis, MO, USA, D8375) was reconstituted in Gibco Water for Injection for Cell Culture (Thermo Fischer Scientific, A1287301) to a concentration of 1 M, aliquoted and stored at −20 °C until used. Liraglutide working stock preparations were diluted in serum-free culture medium to different nM concentrations. In experiments, 2-deoxy-d-glucose was used at 50 mM concentration.

Methylglyoxal solution ~40% in water (Sigma-Aldrich, M0252) working stock preparations diluted in serum-free culture medium to different μM concentrations. In experiments, methylglyoxal was used at 600 μM concentration. 

The concentration of liraglutide, 2DG and MG was selected on the basis of previous experiments by using MTT assay, see Figure S1.

### 4.3. Stable Silencing Pin1 Gene 

Cells were plated the day before the transfection at 80% confluence in 35 mm dishes. Transient transfection was performed using 250 ng of plasmids by using lipofectamine 2000 (Thermo Fischer Scientific, 11668027) following the manufacturer’s instructions. Transient silencing was obtained using two different plasmids either pLKO.1-puro Non-Target shRNA Control Plasmid DNA (Scramble) or Pin1 shRNA (NM_00622_TRCN0000001033; NM_00622_TRCN0000010577 all from Sigma-Aldrich). We got stably transfected clones by using antibiotic selection 1 ug/mL of puromycin (Sigma-Aldrich, P8833) in the culture medium.

### 4.4. Cell Viability Assessment

Cell viability was evaluated by using Cell proliferation Kit I (MTT) (Roche Diagnostics Ltd., Monza (MB), Italy, 11465007001). MTT assay measures cell viability and toxicity based on the cellular reduction of NADH and NADPH. The assays were formatted in Falcon flat-bottomed 96-well plates. The cells were subsequently serum starved in serum-free medium for 5 h and stressed with/without concentrations of 50 mM 2-deoxy-d-glucose for 1 h or 600 μM MG for 24 h, in the presence or absence of 100 nM of liraglutide. All cell treatments were performed in sextuplicate per plate per experiment. The absorbance was measured at 550 and 650 nm (reference wavelength) using a Perkin Elmer Envision 2104 multilabel reader.

### 4.5. Total ATP Content Measurements

ATP levels were assayed luminometrically using the ATPLITE 1 STEP (Perkin Elmer, Waltham, MA, USA, 6016731) according to the procedure recommended by the manufacturer’s instructions. 4 × 10^4^ cells were seeded for 24 h. The ATP content was measured considering the total (10 mM glucose), the glycolytic (10 mM glucose plus 2.5 mg/mL oligomycin; G + O) and the oxidative ATP production (50 mM 2-deoxy-d-glucose plus 5 mM pyruvate; 2DG + P). Glucose (Sigma Aldrich, G8270), Oligomycin A (Sigma Aldrich, 75351), Sodium Pyruvate (Sigma Aldrich, P2256). Briefly, cells incubated for 2 h in record solution accordingly to report elsewhere [43]. Sample’s luminescence was measured using a Perkin Elmer Envision 2104 multilabel reader. 

### 4.6. ROS Production

Intracellular ROS production was determined by chloromethyl-H2DCFDA (Thermo Fischer Scientific, C6827). First, 4 × 10^4^ cells were plated in 96 VIEWPLATE black and flat bottom plates (Perkin Elmer, 6005225) and let growth for 24 h; cells were incubated with 10 uM of CM-H2DCFDA (Thermo Fischer Scientific, C6827) in KREBS-Henseleit buffer (Sigma Aldrich, K3753) for 30 min at 37 °C. ROS production was measured in control conditions and after treatment with or without 2DG, with/without MG in presence or absence of liraglutide treatment. Excitation filter for the CM-H2DCFDA was set at 495 and the emission filter was set at 529 nm. The fluorescence intensity data obtained have been normalized for the cell number using Hoechest 33342 (Thermo Fischer Scientific, H3570) at 350 nm as excitation and 461 nm as emission. The probe’s fluorescence was monitored with a Perkin Elmer Envision 2104 multilabel reader (Perkin Elmer).

### 4.7. Quantitative Real Time-PCR 

Total RNA was extracted from cells using TRI REAGENT (Sigma Aldrich, T9424) following manufacturer’s instructions. The reverse transcription was performed with High-Capacity cDNA Reverse Transcription kit (Thermo Fischer Scientific, 4368814) and 15 ng cDNAs in a 10-μL reaction volume containing 2× SYBR Green PCR Master Mix (Thermo Fischer Scientific, 4309155) were used for the real time PCR reaction. All samples were run in triplicate. The cDNAs were amplified in QuantStudio™ 12K Flex Real-Time PCR System (Thermo Fischer Scientific). All primer were purchased from Sigma Aldrich. Primers list: Pin1-F GACGAGGAGAAGCTGCCGCC; Pin1-R: CAGGCTCCCCCTGCCCGTTT; NRF1-F: CAGCTGCAGGAAACTTCGAG; NRF1-R: CGCACCACATTCTCCAAAGG; PGC-1α-F: GGACTCAAGTGGTGCAGTGA; PGC-1α-R: GTGTCTCTGTGAGGACTGCT; SIRT1-F: GCGATTGGGTACCGAGATAA; SIRT1-R: GTTCGAGGATCTGTGCCAAT; FIS1-F: TACGTCCGCGGGTTGCT; FIS1-R: CCAGTTCCTTGGCCTGGTT; MFN1-F: TGTTTTGGTCGCAAACTCTG; MFN1-R: CTGTCTGCGTACGTCTTCCA; BAX-F: CAGCAAACTGGTGCTCAAGG; BAX-R: GAAGTCCAATGTCCAGCCCA; BCL2-F: GAACTGGGGGAGGATTGTGG; BCL2-R: CATCCCAGCCTCCGTTATCC; CASP3-F: TGCATACTCCACAGCACCTG; CASP3-R: TTCTGTTGCCACCTTTCGGT; TP53-F: GCTTTGAGGTGCGTGTTTGT; TP53-R: AGAGGAGCTGGTGTTGTTGG; β Actin-F: AGACCTGTACGCCAACACAG; β Actin-R: TCTGCATCCTGTCGGCAAT.

### 4.8. Western Blotting 

Cells were resuspended in lysis RIPA buffer (Sigma Aldrich, R0278) with the addition of Halt™ Protease and Phosphatase Inhibitor Cocktail (Thermo Fischer Scientific, 78440). The protein concentration was determined using the BCA protein assay kit (Thermo Fisher Scientific, 23235). Blots were washed once in 1× TBS for 5 min, blocked in 5% *w*/*v* non-fat dry milk for 1 h at room temperature, and probed with the primary antibodies against: Pin1(Santa Cruz Biotechnology, Dallas, TX, USA, sc-46660); β-Actin (Santa Cruz Biotechnology, sc-47778); OPA1 (BD Biosciences, San Jose, CA, USA, 612606); FIS1 (Enzo Life Sciences, ALX-210-907,1:800); DRP1 (DE11B11) (Cell Signaling, Beverly, MA, USA, 14647). Primary antibodies were diluted in 5% BSA in 1X TBS with 0.05% Tween 20: GSK-3-beta (27C10) (Cell Signaling, 9315); Phospho-GSK-3-beta (Ser9) (Cell Signaling, 9323); Phospho-Akt (Ser473) (D9E) (Cell Signaling, 4060); Akt (pan) (C67E7) (Cell Signaling, 4691); phosphoDRP1(Ser616) (Cell Signaling, 3455). Blots were washed three times in 1× TBS–T for 5 min each and incubated with the HRP-linked secondary antibodies against the corresponding species IgG (1:8000) for 1 h at room temperature. Blots were developed using Amersham ECL Prime western blotting detection reagent kit as per manufacturer’s instructions. ChemiDoc™ MP Imaging System with Image Lab™ software (BIO-RAD Laboratories, Hercules, CA, USA) used to image chemiluminescent bands and perform densitometric analysis. β-Actin protein used as loading control to which relative peak intensities of the examined markers were normalized. 

### 4.9. Examination of Apoptosis by Flow Cytometer 

Apoptosis was assessed by FITC Annexin V Apoptosis detection Kit (BD Pharmigen, San Jose, CA, USA, 556547) staining after cells were pre-treated with or without 100 nM liraglutide for 5 h and then exposed in presence or absence of 600 μM MG for 24 h in serum free medium. Briefly, cells were washed in PBS and re-suspended in Annexin Binding Buffer (10 mmol/L HEPES pH 7.4, 140 mmol/L NaCl, and 2.5 mmol/L CaCl). Cells were then stained with 0.5 mg/mL Annexin V-FITC/Propidium iodide for 15 min in the dark before analyzing. Finally, 400 μL of 1× binding buffer was added to each tube, and the frequency of apoptosis was analyzed by flow cytometry within 60 min. Acquisition and analysis were carried out on a Becton Dickinson FACSCanto II flow cytometer (Becton-Dickinson, Milan, Italy), using DiVa Software, version 6.3 (Becton Dickinson).

### 4.10. Immunocitochemistry

Parental SH-SY5Y cells and stable clones were seeded on glass coverslips for 24 h. Cells were pre-treated with or without 100 nM liraglutide for 5 h and then exposed in presence or absence of 50 mM 2DG for 1 h in serum free medium. Cells were fixed in 4% paraformaldehyde, and then permeabilized with 0.1% Triton X-100 in PBS. Cells were incubated in blocking buffer (10% normal goat serum in PBS) for 2 h and then incubated over-night with the following primary antibodies: anti-Pin1 (Santa Cruz Biotechnology, sc-46660, 1:100,) and anti-TOM20 (Santa Cruz Biotechnology, sc-11415, 1:500). Subsequently, cells were incubated with the secondary (1:500), Alexa Fluor 488 (Thermo Fischer Scientific, A11070) and 555 (Thermo Fischer Scientific, A21425), and, after a brief wash, Hoechst 33342 (Thermo Fischer Scientific, H3570) were used to counterstain for the nucleus. Fluorescence images were acquired using Olympus Fluoview FV1000 confocal microscope equipped with FV10-ASW version 4.1 software, using 60× (1.42 numerical aperture) oil objective. Z-reconstructions of serial single optical sections were acquired with a 1024 × 1024 format, scan speed of 400 Hz, a pixel size of 0.1 μm, and z-step size of 0.3 μm. Lasers’ power, beam splitters, filter settings, pinhole diameters and scan mode were the same for all examined samples of each staining. To improve contrast and resolution of confocal raw images, deconvolution analysis (Huygens Essential software, Scientific Volume Imaging, Hilversum, the Netherlands) was applied to Z stacks images. The average intensity of TOM20 fluorescence was calculated using FV10-ASW software, from cytometric measurements relative to cell area positive to TOM20 antibody in 10 confocal images randomly selected and analyzed for each cell sample. Hundred to 140 cells were examined for each sample analyzed.

### 4.11. Statistical Analysis

Data are presented as mean ± SD. The Student’s *t*-test was used for the analysis of statistical significance. A *p* value < 0.05 was considered significant. 

## 5. Conclusions

We developed an engineered neuronal model that might mimic impaired energy metabolism and mitochondrial involvement of neurons in patients with T2D associated dementia or AD. Silencing of Pin1 produced increased cell energy metabolism, enhanced mitochondrial fission, ROS production and susceptibility to apoptosis in presence of MG. Treatment with 100 nM Lira rescued some of the metabolic dysfunctions induced by Pin1 silencing and treatment with 2DG or MG.

We are aware that findings of the present investigation are far from being transferable from the bench to the bedside, at least at this time. First, they have been produced in neuroblastoma cells whose metabolism and cell responses might differ from those of primary neuronal cells. Nevertheless, SHSY5Y cells are widely used as an in vitro model of neurons. Pathogenesis and Pin1 mediated mechanisms behind progression of altered brain energy metabolism to neurodegeneration in humans are expected to be more complex involving different cell types (i.e., glial cells) in a network; varying among the different brain areas (i.e., cortical versus non-cortical) and along the history of disease (i.e., prodromal phase, onset and progression). The role of Pin1 in neuronal energy metabolism deserves investigation in robust in vivo models. Secondly, we treated engineered cells with liraglutide 100 nM that corresponds to a concentration of 0.37 mg/L, while the dose for clinical use ranges from 0.6 to 1.8 and 3.0 mg per day in patients with T2D and obesity, respectively. An investigation in patients with T2D demonstrated a very low concentration of GLP-1 analog in the cerebrospinal fluid of treated patients suggesting its reduced passage across the brain blood barrier. Nevertheless, the concentration reached in the brain seems sufficient to induce a clinical effect [68].

## Supplementary Materials

Supplementary materials can be found at https://www.mdpi.com/1422-0067/20/20/5064/s1. Figure S1A: Cell viability of SH-SY5Y cells exposed at different concentrations of Lira (0, 50, 100, 200 nM) for 5 h. Figure S1B: Evaluation of cell toxicity of SH-SY5Y cells to 2DG treatment. Cells were starved in serum free medium for 5 h then SH-SY5Y cells were exposed to different concentrations of 2DG (0, 25, 50, 100 mM) for 1 h. S1C. Evaluation of cell toxicity of SH-SY5Y cells to MG treatment. Cells were starved in serum free medium for 5 h then SH-SY5Y cells were exposed to different concentrations of MG (0, 300, 600 μM) in serum free medium for 24 h.

## Abbreviations

AD	Alzheimer Disease
AGEs	Advanced Glycation End-Products
AMPK	AMP-activated Protein Kinase
APP	Amyloid Precursor Protein
Aβ	Amyloid beta
ATP	Adenosine Triphosphate
BAX	BCL-2 associated X
CASP3	Caspase3
2DG	2-Deoxy-D-Glucose
DRP1	Dynamin-Related Protein 1
pDRP1(S616)	PhosphoDynamyn Related Protein 1(S616)
FIS1	Fission1
G	Glucose
GLP-1	Glucagon-Like-Peptide1
GSK3β	Glycogen Synthase Kinase-3β
pGSK3β	PhoshoGlycogen Synthase Kinase-3β
Lira	Liraglutide
MFN1	Mitofusin 1
MG	MethylGlioxal
NRF1	Nuclear Respiratory Factor 1
O	Oligomycin
OPA1	Optic Atrophy 1
P	Parental cells
P1-A7	Pin1 KD
P1-C7	Pin1 KD
Pin1	Peptidylprolyl cis/trans isomerase NIMA-interacting 1
Pin1 KD	Pin1 knock down
PI3K	Phosphoinositol 3 kinase
PGC1α	Peroxisome proliferator-activated receptor gamma coactivator 1-alpha
qPCR	Real-time quantitative PCR
ROS	Reactive Oxygen Species
Sc	Scramble
SIRT1	NAD-dependent deacetylase 1 Sirtuin 1
T2D	Type 2 Diabetes
T3D	Type 3 Diabetes
