# Supplementary figures and images for: Liraglutide Treatment Ameliorates Neurotoxicity Induced by Stable Silencing of Pin1
Source: Int J Mol Sci. 2019 Oct 12;20(20):5064. doi: 10.3390/ijms20205064 (PMC6829573; doi:10.3390/ijms20205064)

**A**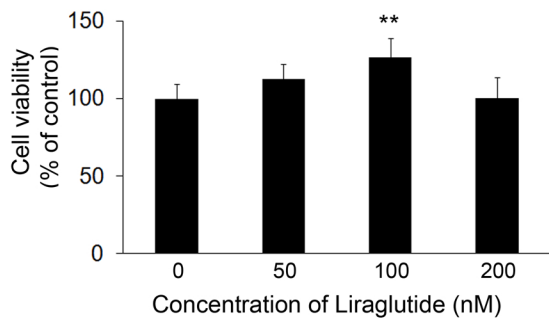**B**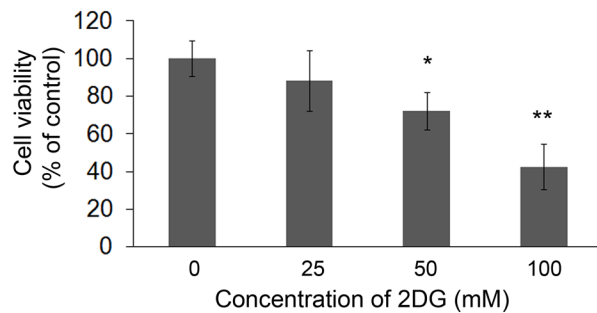**C**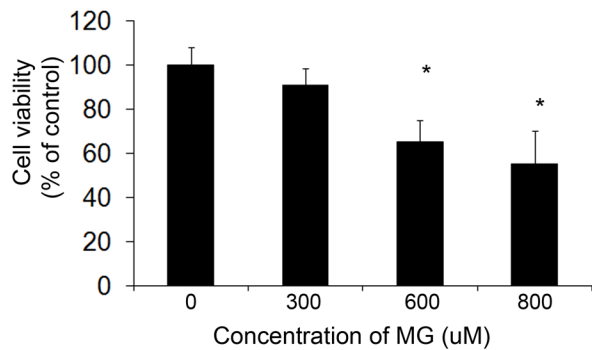

Supplement: Supplementary file 1 [file ijms-20-05064-s001.pdf]
